# Improving Pediatric Cancer Care Disparities Across the United States–Mexico Border: Lessons Learned from a Transcultural Partnership between San Diego and Tijuana

**DOI:** 10.3389/fpubh.2015.00159

**Published:** 2015-06-22

**Authors:** Paula Aristizabal, Spencer Fuller, Rebeca Rivera, David Beyda, Raul C. Ribeiro, William Roberts

**Affiliations:** ^1^Division of Pediatric Hematology/Oncology, Department of Pediatrics, Peckham Center for Cancer and Blood Disorders, Rady Children’s Hospital San Diego, University of California San Diego, San Diego, CA, USA; ^2^Reducing Cancer Disparities Program, University of California San Diego Moores Cancer Center, La Jolla, CA, USA; ^3^University of California San Diego School of Medicine, La Jolla, CA, USA; ^4^Pediatric Hematology/Oncology, General Hospital de Tijuana, Tijuana, Mexico; ^5^Global Health Program, University of Arizona College of Medicine, Phoenix, AZ, USA; ^6^Department of Oncology, St. Jude Children’s Research Hospital, Memphis, TN, USA; ^7^University of California San Diego Moores Cancer Center, La Jolla, CA, USA

**Keywords:** pediatric cancer, transcultural partnership, US–Mexico border, international oncology, health systems strengthening, global health, border health

## Abstract

In 2007, the 5-year survival rate for children with acute leukemia in Baja California, Mexico was estimated at 10% (vs. 88% in the United States). In response, stakeholders at St. Jude Children’s Research Hospital, Rady Children’s Hospital San Diego, and the Hospital General de Tijuana (HGT) implemented a transcultural partnership to establish a pediatric oncology program. The aim was to improve clinical outcomes and overall survival for children in Baja California. An initial needs assessment evaluation was performed and a culturally sensitive, comprehensive, 5-year plan was designed and implemented. After six years, healthcare system accomplishments include the establishment of a fully functional pediatric oncology unit with 60 new healthcare providers (vs. five in 2007). Patient outcome improvements include a rise in 5-year survival for leukemia from 10 to 43%, a rise in new cases diagnosed per year from 21 to 70, a reduction in the treatment abandonment rate from 10% to 2%, and a 45% decrease in the infection rate. More than 600 patients have benefited from this program. Knowledge sharing has taken place between teams at the HGT and Rady Children’s Hospital San Diego. Further, one of the most significant outcomes is that the HGT has transitioned into a regional referral center and now mentors other hospitals in Mexico. Our results show that collaborative initiatives that implement long-term partnerships along the United States–Mexico border can effectively build local capacity and reduce the survival gap between children with cancer in the two nations. Long-term collaborative partnerships should be encouraged across other disciplines in medicine to further reduce health disparities across the United States–Mexico border.

## Introduction

According to the World Bank, Mexico was the 11th most populated country in the world in 2011 with roughly 122,330,000 citizens, and spent $620 US per capita on healthcare [6.2% of its gross domestic product (GDP)], ranking 70th worldwide. Based on published data, Mexico’s health outcomes seem to underperform its healthcare expenditure rankings and GDP earnings, with some healthcare outcomes ranking low, such as infant mortality rate (97th), under-five mortality rate (87th), and access to improved sanitation facilities (104th) (1).

In contrast to Mexico, the United States (US) is the third highest healthcare spender at $8,895 US per capita (17.9% of its GDP – the most of any country) (1). While increasing healthcare expenditures does not necessarily correlate with better outcomes, significant health disparities exist between the US and Mexico. Nowhere are these disparities more obvious than in the 47 border-straddling metropolises along the 1,989 mile-long US–Mexico border.

Two of these border cities, San Diego, California, and Tijuana, Baja California (a state also known as “Baja California Norte”), share a 24 km-long border. Close to 60 million people cross this border annually; therefore, it is the busiest land-border crossing in the world. Two of the largest hospitals that provide care to patients in these cities are Rady Children’s Hospital San Diego (RCHSD), the largest children’s hospital in California and the sixth largest children’s hospital in the US, and the Hospital General de Tijuana (HGT), the largest public hospital in northwestern Mexico. These two institutions are separated by only 43 kilometers – a short 30-minute drive – and although the international boundary is very distinct, this border region is culturally, linguistically, demographically, and economically blurred.

With more than 160,000 new cases of childhood cancer diagnosed annually worldwide, the survival gap for children with cancer in high-income countries (HIC) and low- or middle-income countries (LMIC) is astounding (2, 3). Although we recognize that grouping low- and middle-income countries together can be problematic in making comparisons among diverse nations, which include recently emerging economies such as Mexico, we are grouping LMIC together in this report, as in published, related literature elsewhere, due to the fact that these countries tend to have similarly poor outcomes for children with cancer.

Five-year overall survival rates for children with cancer in HIC, like the US, are often 80–90% as compared to anywhere between 5 and 60% for children in an LMIC like Mexico (2–7). This survival gap reflects the great disparities that exist between the healthcare infrastructures of these two categories of countries regarding effective pediatric cancer care.

Mortality due to cancer constitutes an increasingly large proportion of total childhood mortality in LMIC (8–10). Mexico is no exception, and although the incidence of cancer has increased from 150.3 per million children per year in 2010 to 156.9 in 2012, the mortality rate has been reported as 5.3 per 100,000 children and as high as 8.6 in adolescents (11, 12). Reasons for increased morbidity and mortality related to childhood cancer in Mexico and other LMIC are described in Table 1 (12, 13). In these countries, pediatric cancer mortality represents a significant source of largely preventable deaths. As the proportion of deaths due to communicable diseases decreases, the proportion of deaths due to cancer increases (2, 13).

**Table 1:** **Differences in pediatric cancer care between high-income countries (HIC) and low- or middle-income countries (LMIC)**.

Feature	HIC – 20% of population	LMIC – 80% of population
Access to Care	Virtually 100%	10–70%
Causes of treatment failure	Relapse	Late referrals
	Toxicity Cancer resistance	Advanced disease at presentation
		Abandonment of treatment
Major current focuses	Finding cures	Increasing access to care
	Improving quality of life posttreatment	Improving survival Reducing suffering
Activities	Discovering disease mechanisms	Preventing abandonment
	Development of targeted therapies Mitigating long-term complications	Educating the community Adapting curative therapy to local resources and populations

Given the need for improved pediatric cancer care in the Mexican border region and the proximity to San Diego, leaders at RCHSD reached out to numerous hospitals in Tijuana to assess interest in engaging in a binational, collaborative partnership to establish a pediatric oncology unit in Tijuana and improve outcomes for children with cancer. Among the institutions contacted, the HGT responded and expressed its willingness. Subsequently in 2008, RCHSD, the St. Jude Children’s Research Hospital International Outreach Program (St. Jude IOP), and the HGT engaged in this long-term partnership to find a sustainable, local solution for the children with cancer of Baja California in need of life-saving oncology care. To the best of our knowledge, as of 2008, a transcultural partnership in pediatric oncology had not yet been established between institutions located in such close proximity of the same border.

## Materials and Methods

### Implementation: Initial setting and action plan

Physicians from RCHSD performed an initial site visit assessment at the HGT in May 2008. The St. Jude IOP had previously developed tools to aid in performing needs assessments (14). One of these tools was adapted to the local setting in Tijuana to include elements needed to obtain national accreditation from the Mexican Ministry of Health. (This tool and budgetary guidelines can be requested from the corresponding author.) The assessment analysis showed that, even though the hospital had a basic infrastructure, the essential elements of a pediatric cancer unit, including dedicated hospital space, uniform treatment protocols, supportive care, trained physicians, nurses, and allied staff, were lacking.

A bilingual and bicultural pediatric hematologist/oncologist from RCHSD (PA) was designated to work with the leadership at the HGT and designed a 5-year action plan to facilitate the implementation of the new pediatric oncology program. PA worked in close collaboration with RCHSD and St Jude IOP leadership. In addition, she directly interacted with hospital leadership and key stakeholders at the HGT at all levels. PA monitored the progress by close collaboration and follow-up with all key stakeholders. Careful attention was made to the suggestions and guidelines previously published in the literature in regards to transcultural partnership implementation (3, 10, 15–17) and the Health Systems Strengthening principles were followed (18). The specific objectives of the St. Jude IOP-RCHSD-HGT partnership became the following:

1. Infrastructure and clinical outcomes improvement by establishing a fully functional pediatric oncology unit within the HGT in a culturally sensitive manner and tailored to the local needs and healthcare system and developing a quality assurance plan.
2. Capacity building by appointing a local program coordinator at the HGT, maintaining frequent communication between RCHSD and the HGT, and establishing and training a local, dedicated team of physicians, nurses, and ancillary staff to provide high-quality, specialized care.
3. Ensuring the long-term financial sustainability and provision of medications, supplies, and equipment by facilitating the pediatric oncology unit’s national accreditation and identifying and supporting a local grassroots foundation affiliated with the HGT to build a fundraising program aimed at subsidizing the cost of expenses not covered by the public health system.

## Results

### Infrastructure and clinical outcomes improvements

#### Infrastructure Improvements

Before the partnership, it was not uncommon for the HGT to assign 10–20 pediatric patients to a single room on the pediatric ward. By August 2008, construction of a temporary pediatric oncology unit with five rooms was completed. In this temporary unit, cancer patients were able to receive care in an isolated environment. The current oncology unit in use today was constructed in 2011 and includes 10 isolated patient rooms, two intensive care rooms, and adequate bathroom facilities; an outpatient clinic was also completed with a procedure room, clinic rooms, an infusion center, and a school for the children.

Table 2 shows some of the clinical workflow enhancements and quality assurance components that were implemented in order to improve the delivery of clinical care in these new facilities.

**Table 2:** **Clinical workflow improvements and quality assurance components at the Hospital General de Tijuana pediatric oncology unit**.

Adjustments to the process for lab draws
Provision of vital equipment and utilities: computers, internet, and phone service
Process optimization regarding medication administration
Reorganization of the space in the outpatient clinic and infusion center
Medical record documentation and establishment of a medical record archive
Development of local institutional guidelines and protocols, an institutional cancer registry and a data management program

Four factors were essential to the infrastructure developments:

1. The creation of a start-up budget between St. Jude IOP, RCHSD and the HGT to provide initial funding.
2. Assisting the HGT with its application for national accreditation from the Mexican Ministry of Health. Such accreditation ensured that the HGT would receive future funding from Seguro Popular.
3. The close working relationship between key stakeholders at RCHSD and at the HGT to create a large-scale, feasible plan.
4. The extra $1 million US provided by the Mexican federal government to enhance the infrastructure at the HGT.

#### Patient Clinical Outcomes

The patient population tripled soon after the inception of the program. Leukemia, lymphoma, and brain tumors have constituted the most common cancers diagnosed in this population, and the disease breakdown seems to be a representative sample of the tumor burden in Mexico (11, 12) as well as in HIC (19). Significant improvements in patient outcomes are the direct result of the establishment of a fully functional pediatric oncology unit, a dedicated team that has worked to improve access to care for those underserved patients who previously could not have afforded it, the establishment of infection management, supportive care, and transfusion protocols, and the implementation of a data management program that has subsequently been used to guide clinical decision making.

**Figure 1:**
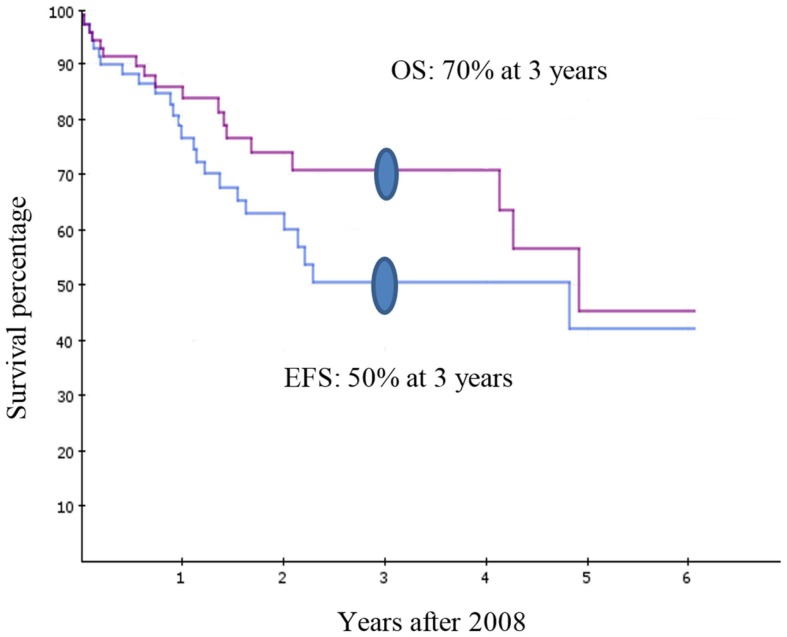
**Kaplan–Meier overall survival (OS) and event-free survival (EFS) curves for the patients with acute lymphoblastic leukemia at the Hospital General de Tijuana pediatric cancer unit from 2008–2014**.

Figure 1 includes the Kaplan–Meier curves for overall survival and event-free survival for patients treated at the HGT cancer unit from 2008 to 2014. The 3-year overall survival rate for acute lymphoblastic leukemia achieved at the HGT in less than 4 years (from 2008 to 2011) was 70% and the event-free survival was 50%, remarkable feats in such a short-time period.

Table 3 includes other patient outcome accomplishments at the HGT. For example, deaths related to infections have dropped dramatically from 25% in 2008 to 0% in 2013. Also, there has been a 45% reduction in the infection rate over the same period. In regard to the highly curable cancers like acute lymphoblastic leukemia and lymphomas, the leaders of the program on both sides of the border expect to achieve similar survival rates as those for children in San Diego by 2020.

**Table 3:** **Selected clinical outcomes in pediatric oncology at the Hospital General de Tijuana**.

Category	2008	2014
New cases per year	21	70
3-year survival rate for acute lymphoblastic leukemia	10%	70%^c^
Relapse rate	Unknown	22%
Abandonment rate	10%	2%
Mortality rate during induction therapy	Unknown	5.3%^a^
Infection rate (# infections × 100/inpatient days–month)	3.3	1.8
Infection-related mortality rate	27%	0%^b^

*^a^Higher rate when compared to institutions in high-income countries, but lower when compared to other low- or middle income countries (20, 21)*.

*^b^Data from 2013*.

*^c^Data from 2011*.

### Capacity building

#### Team Building Accomplishments

The establishment and training of a multidisciplinary healthcare team is one of the most effective ways to achieve a high-quality pediatric cancer program where resources are constrained (10, 16, 22), and the same has held true for the St. Jude IOP-RCHSD-HGT partnership. A local pediatric oncologist was appointed as the on-site coordinator to oversee the emerging projects. Table 4 reports the enhancements made to the team makeup as viewed by snapshots taken in 2007, 2008, and currently in 2015 that display the type and number of healthcare professionals employed by the HGT pediatric cancer unit in those years.

**Table 4:** **Healthcare professional development progress at the pediatric oncology unit, Hospital General de Tijuana, 2007–2015**.

Position	2007 (*N* = 5)	2008 (*N* = 27)	2015 (*N* = 60)
Pediatric oncologists	0	1	3^a^
Pediatric hematologists	0	0	1^a^
Pediatric intensive care specialists	0	1	1
Anesthesiologists	0	0	1
Pediatric infectious disease specialists	1	1	1^a^
Trained pediatricians	0	6	8^a^
Nurses	1	10	31^a^
Psychologists	0	1	3^a^
Social workers	1	1	1^a^
Dieticians	0	1	1
Pharmacists	0	1	1^a^
Teachers	0	0	2^a^
Pathologists	1	1	1
Surgeons	1	1	1
Administrative assistants	0	1	2^a^
Ambulance drivers	0	1	1^a^
Data managers	0	0	1^a^

*^a^Exclusive to the Hospital General de Tijuana pediatric oncology unit*.

#### Training

Training for the physicians, nurses, and ancillary staff, including social workers, psychologists, and data managers, has been one of the pillars of this project. Training has taken place on-site and in other partner sites affiliated with the St. Jude IOP, such as Guatemala and Chile (3, 8). For the staff fluent in English and able to cross the border, training has been conducted at RCHSD. More than 6,000 hours of on-site and online training have been provided to various staff members. Patients and families have also benefited from education and counseling about nutrition, oral health, and infection prevention.

One initial problem was the scarce number of pediatric oncologists trained to care for children with cancer. A “physician-extender” model was used as a solution, where a physician with training in one area pursues additional, specialized training to expand the care he or she can provide (23). Within the HGT’s pediatric cancer unit, there are currently eight general pediatricians who obtained further pediatric oncology and infectious diseases training to work as “physician-extenders” to provide specialized care to critically ill patients.

Community outreach education and cancer workshops to educate local and regional healthcare providers and the general public about pediatric cancer have been one of the principal goals of the educational program. To date, nearly 2,500 community health care providers in Baja California have been reached by this initiative.

Each of these training efforts has notably changed the skill level, dynamic, and culture of the multi-disciplinary team. Physicians are now well-trained to care for patients with cancer; nurses now feel respected as vital members of the team; and doctors and nurses now work together with other professionals and provide standard of care to their patients. A powerful change has taken place within a relatively short period of time.

### Financial sustainability

#### National Accreditation

One of the priority action items since the planning stages of the program has been the facilitation of the national accreditation to ensure federal financing. None of the accomplishments related to infrastructure and clinical outcomes would have been possible without the constant funding provided by the Mexican national government through Seguro Popular. Seguro Popular provides a fixed amount of monies per patient per year based on the diagnosis. For example, for a patient with acute lymphoblastic leukemia, Seguro Popular disburses to accredited centers about $10,000 US annually. This fund covers for the majority of medications, including chemotherapy and antibiotics, supplies, and direct hospital costs. The local state government through its Ministry of Health supports the salary for personnel, equipment, and additional operational costs.

This model has ensured a stable and predictable funding stream to provide comprehensive care for pediatric cancer patients, an essential element to the long-term success of any health system’s strengthening effort (18).

#### Grassroots Foundation

Despite the immense financial contributions made by Seguro Popular, the funds have not been sufficient to meet the HGT’s needs. Therefore, local grassroots efforts have offered immense support to the patients and their families. Patronato Pro-Hospital de Tijuana – Patronato – is a local, apolitical organization founded in 2000 to support all patients at the HGT, which are often vulnerable. Given that the HGT is the largest public, community hospital in Northwestern Mexico, it serves as a safety net for the poorest in Baja California; as a result, nearly all families that seek care at the HGT live below the effective poverty line of $200 US per month. Among other services, Patronato provides food subsidies, funding for some medications not covered under Seguro Popular and lodging for families who reside outside Tijuana (about 35% of families). This strategy facilitates access to care and helps prevent abandonment of treatment. Thus, without Seguro Popular and the philanthropic efforts of Patronato, patients at the HGT could not have access to treatment and would more frequently be lost to treatment abandonment, since the majority of patients receiving care at the HGT cannot afford the high expenses associated to oncology care, including the cost of travel to the HGT, food, missing work for one or both parents to support their child, and many other expenses. Since the inception of the partnership program, cancer care has been provided free of all costs to all patients younger than 18 years of age receiving care at the HGT regardless of their income or socio-economic status. Moreover, Patronato has been the vehicle for the disbursement of funds provided by the St. Jude IOP-RCHSD partnership to sponsor all training activities and salary supplementation for key personnel at the HGT.

In addition, close relationships between leadership of the partnership program and Patronato have led to effective community advocacy contributions to strengthen relationships with local government leaders. This focus has been instrumental to the successful launching and sustainability of the program.

## Discussion

Collaborative partnerships in pediatric oncology between institutions in HIC and LMIC may overcome health disparities by strengthening health systems and improving clinical outcomes for children with cancer. Innovative and dynamic collaborative models, where an academic pediatric oncology center in a HIC establishes a long-term partnership with an emerging center in a LMIC have been successful in improving survival rates for children with cancer (13). One of such programs was established in 1986 between La Mascota Pediatric Hospital in Managua, Nicaragua, and hospitals in Monza and Milan, Italy, and Bellinzona, Switzerland, with successful outcomes (10). Since then, the Monza International School of Pediatric Hematology/Oncology and the St. Jude IOP have become pioneers in facilitating new collaborative programs throughout the world. To date, the St. Jude IOP has established more than 20 programs in 15 countries. As a result, 5-year survival rates for children with cancer have significantly increased in the regions where these partnerships have been established (8, 15, 22), particularly for children who suffer from acute lymphoblastic leukemia. In addition, these programs help develop and strengthen local healthcare infrastructures in comprehensive, sustainable ways from the ground up (2, 8). These partnerships are often based on proven Health Systems Strengthening principles, like holism, context and collaboration, social mobility, capacity enhancement, equity, financial protection, and evidence-informed action (18).

### The ethics of transcultural partnerships in healthcare

Numerous ethical principles have been at work throughout the successful St. Jude IOP-RCHSD-HGT collaborative initiative. In general, transcultural partnerships elicit several ethical issues that should be addressed prior to engaging in the relationship. They are not necessarily the classical principles of ethics such as beneficence, maleficence, justice, or autonomy, but center around an understanding of intercultural aspects and communications. There should be an understanding of the dynamics between each party with sensitivity to specific cultural norms and behaviors. There can be ethical conflicts that relate to relationships and trust. Is the trust based on signed agreements or does it depend on the relationship between the two parties? If the trust is based on relationship, when and how does the relationship begin and end?

One also needs to be aware of the differences between autonomous decision making and paternalism. One partner should not expect to come in and direct decisions. Rather, all parties involved should guide each other and have a common motivation, similar perspectives, and unifying goals. Specific ethical issues will include potential sources of conflict such as emotions, assumptions and perceptions, values, needs and goals, lack of information or clarity, and individual styles of communication and behavior. To achieve a successful working relationship, stakeholders would benefit by separating any conflicts from concepts of right and wrong, and considering looking at or doing things in different ways that would be acceptable to all parties.

### Overcoming disparity in mexico

Health disparities in pediatric oncology are well-documented, as are the epidemiology and burden associated with childhood cancer in developing countries (4, 13–15).

In 2012, the Mexican census reported 32,972,300 children aged 0–18 years, comprising 37% of the total population (11). Of those, less than 8% are covered with commercial insurance and around 40% have access to healthcare through their employed parents who are insured via social security programs (Instituto Mexicano de Seguridad Social-IMSS). The overwhelming majority – over 50% – are uninsured children, whose parents are not salaried workers, are rural residents, or unemployed. All Mexicans who are uninsured are eligible for Seguro Popular coverage (11, 12).

In Mexico, cancer is the sixth leading cause of death in children 1–4 years old and the second leading cause of death in children 5–14 years old (11, 12). Prior to 2008, outcomes were very poor for children with cancer in Baja California (20, 21, 24), and because a comprehensive pediatric cancer center did not exist in Baja California at that time, newly diagnosed pediatric oncology patients from the region traveled long distances to other hospitals in Mexico to seek care, or sadly, died in their local communities (20, 24). Some reasons why pediatric cancer care was still poor in Baja California as compared to other regions of central Mexico were because of the high poverty rate of the citizens of Tijuana (25), the relative insufficient number of academic institutions in Baja California, the scarcity of philanthropic initiatives supporting patients with chronic diseases, and the geographic isolation that characterizes states not located on central Mexico (26–28). In addition, the Seguro Popular program created to cover for oncology care only started in Mexico in 2007.

The economic differences between the US and Mexico have contributed in part to some of the healthcare disparities that extend into the discipline of pediatric oncology. Before 2008, there was neither a pediatric oncology unit nor a pediatric oncologist in all of Baja California. Lack of pediatric cancer care is a problem that has extended beyond the San Diego–Tijuana border. In 2012, Mexico had 3.6 pediatric oncologists per million children under the age of 18 (2), while the US had 29.2 total pediatric cancer specialists per million children (3). Large regional staffing inequalities exist in Mexico (4), as 44% of oncologists are concentrated in Mexico City, and 18% are in Guadalajara and Monterrey, the second and third largest cities (11, 12). On average, a pediatric oncologist in Mexico provides care to 65 new cancer cases per year (11, 12), whereas in the US, an oncologist provides care to 10 new cases annually (29).

The Mexican government acknowledged improvements needed to be made to its healthcare system, including improvements in pediatric cancer care; thus in 2006, the highly-acclaimed Seguro Popular legislation was passed. Seguro Popular is more properly known as the System of Social Protection for Health, and within it exists the Fund for Protection against Catastrophic Expenditures that specifically covers pediatric oncology medical care. The Fund for Protection against Catastrophic Expenditures was expanded in 2008 to include full payment for all types of childhood cancers (9, 12, 24).

These initiatives targeted to Mexican children with cancer represent a major effort toward providing appropriate care for these traditionally underserved patients and represent a model for other LMIC across the world (9, 24, 30).

### Program success based on the health systems strengthening principles

In addition to recognizing cultural differences in the St. Jude IOP-RCHSD-HGT partnership, we employed several guiding Health Systems Strengthening principles as outlined by Swanson et al. (18) during the implementation of the program. These principles include:

1. Holism: One takes a holistic approach when one considers all systems components, processes, and relationships simultaneously. A successful transcultural partnership ultimately succeeds based on a true bilateral relationship, one where both institutions work through and improve the processes of the new oncology unit. Leaders at RCHSD took a holistic approach with the HGT by making at least weekly site visits from 2008 to 2010 and monthly visits afterward and by serving as accessible day-to-day mentors as problems arose.
2. Context and collaboration: One considers the context when one considers global, national, regional, and local culture and politics. Collaboration takes place when long-term, equal, and respectful relationships are fostered between mentors and mentees, in all sectors and organizations involved. Since the inception of the program, one of the priorities has been to foster an equal, long-term relationship with the leadership at the HGT, the state government, and the healthcare team in Tijuana. This spirit of cultural and political sensitivity in relationships with Mexico’s Secretary of Health, the HGT leadership, local staff, and patients has been crucial to the success of various aspects of the program.
3. Social mobilization: Social mobilization refers to efforts that advocate for social and political change to improve health systems and social determinants of health. In Tijuana, Patronato has played a major role, as the main advocate of the children, impacting the local community and society and leading to a successful development of the new HGT pediatric cancer program.
4. Capacity enhancement: This occurs when individuals, communities, ministries of health, and other stakeholders in the health system become better able to provide for the various levels of healthcare and accept ownership for doing so. This is one of the greatest strengths of this particular partnership, as capacity enhancement has taken place at all levels, such that there now exists an independently operating pediatric cancer unit in Baja California for the first time. The Secretary of Health has become more engaged in supporting the development of pediatric cancer services in Mexico, and community pediatricians and primary care providers in Baja California are better able to diagnose cancer and refer patients promptly to the HGT.
5. Equity: One strengthens health systems in an equitable way when one focuses on and empowers those who are marginalized. The very purpose of this transcultural collaboration is to reduce health disparities for children with cancer in the border region and to improve access to care and, ultimately, clinical outcomes. This mission continues to expand as the HGT has become a regional referral center and agent of healthcare change. The HGT recently has begun mentoring the Hospital General de La Paz, Baja California Sur, Mexico, as they seek to establish the first pediatric cancer unit in the state of Baja California Sur.
6. Financial protection: Predictable funding streams dramatically affect the way an organization is able to operate and plan. The initial shared and realistic budget between St. Jude IOP, RCHSD, and the HGT was vital to the long-term planning, local sustainability, and success of the partnership. Specific training in budgeting and management RCHSD physician leaders had received prior to the partnership was invaluable. Predictable funding through Seguro Popular has been crucial to the success and sustainability achieved thus far.
7. Evidence-informed action: This is when systems and processes are put in place to gather, analyze and apply local data, make decisions based on evidence, monitor programs, and strive for transparency. The HGT has implemented a new data management system that allows for analyses of data and helps with decision-making activities. Also, the transcultural partnership process was guided according to previously established best practices from earlier collaborative programs and the team at the HGT now has access to treatment protocols and therapy guidelines already developed at RCHSD and St. Jude IOP.

### Challenges

The establishment of this partnership has faced some challenges, including cultural differences between the RCHSD and the HGT teams. Appointing a bilingual and bicultural physician from RCHSD (PA) to serve as the project medical director was the first step in overcoming those barriers. More effective communication in the language spoken at the partner site facilitated mutual understanding. The net result of this dynamic was that the HGT hospital administrators, physicians, nurses, and staff were empowered to take ownership of the evolving cancer program.

A crucial element of the new unit that required creativity and commitment was the constant adaptation of the delivery of care by the HGT staff to the constantly changing amount of resources available to them; during these uncertain times, it was important to find ways to move forward and make improvement, and not wait for situations outside the hospital to change (10).

Despite their commitment to the cause, individual healthcare workers still face their own limits while caring for children with complex, life-threatening treatment complications. These professionals often have a high work load, may receive lower salaries, and often feel undervalued. Employee motivation and morale can suffer as a result. One of the solutions has been the implementation of innovative incentive programs for nurses and allied staff to reward their efforts to provide excellent care.

A limitation of the interpretation of our results is that we were not able to compare the survival rates at the HGT with other similar pediatric oncology units in Mexico in an individualized manner or to those obtained in well-established pediatric oncology centers such as Instituto Nacional de Pediatria, Hospital Infantil de Mexico, or Instituto Mexicano de Seguro Social, given the scarcity of published data in the field (24). In addition, there is no national cancer registry in Mexico; however, there are emerging efforts that started in 2007 to obtain epidemiological data at least for the cases identified through the Seguro Popular system (11, 12). Moreover, we could not assess differences on clinical outcomes in private institutions such as Hospital ABC or Hospital Angeles given that only 5–7% of children in Mexico are privately insured, thus data on these patients would be insufficient for meaningful comparisons.

### Knowledge sharing

Engaging in this transcultural partnership has not only benefited cancer patients in Baja California. As stakeholders of the St. Jude IOP-RCHSD-HGT partnership learned a unique body of knowledge, such as techniques to reduce treatment abandonment rates in Tijuana, physicians in San Diego applied those principles to help with their patients with high risk for abandonment of therapy among their own underserved Hispanic population. Abandonment is not a problem frequently encountered by pediatric oncologists in the US, thus providers often lack the training to deal with families, especially large, involved Hispanic families, when this difficult situation arises. The team in San Diego has learned from the team in Tijuana to adequately address issues with these families in a culturally sensitive and tailored way; they are now cognizant of and respectful of their specific beliefs and Hispanic family values.

In addition, when made aware of the realities faced in resource-restrained settings through the close working relationships with the pediatric cancer service in Tijuana, the team and leaders in San Diego have grown to better appreciate the precious and sometimes perceived as “unlimited” resources in the US. This realization has resulted in more cost-conscious medical care by the team at RCHSD.

### Going forward

Despite all these improvements, 50% of the total expected number of new cases of pediatric cancer in Baja California are still not diagnosed or treated in the current pediatric cancer unit at the HGT. The distribution of the pediatric cancers currently treated at the HGT mimics the epidemiology of pediatric cancers in other areas of the world (11, 12, 19). According to recent Mexican Census data, one would expect about 200 new pediatric cancer cases per year in Baja California (7, 31). There may actually be even more annual cases because about 130,000 new immigrants move to the state each year and thus Census population data may not reflect these additional inhabitants.

Based on data obtained from the HGT’s newly established institutional registry between 2008 and 2014, the number of new cases per year has risen from about 20 to about 70, which is the number of expected cases solely for the population of Tijuana. About 35% of patients treated at the HGT come from outside the city, so while access to care has increased for children with cancer in Tijuana and Baja California, there are still children both in the city of Tijuana and in the state who are not receiving the life-saving care they need.

Looking to the future, efforts will concentrate around improving further the care, the infrastructure, and promoting awareness of pediatric cancer in Baja California. The community outreach programs will continue to train pediatricians, primary care providers, and health “promotores” throughout the state in order to prepare them to detect pediatric cancer earlier in communities state wide.

Initiatives going forward include:

1. Completing the HGT pediatric cancer unit’s transformation into a regional referral center that provides high-quality pediatric oncology care in Mexico and becoming a mentor for the Hospital General in La Paz, Baja California Sur and other hospitals in the region.
2. Furthering innovations to retain the nursing staff and other healthcare workers and keep them motivated.
3. Continuing to grow the “physician–extender” training and retention program, with the hope that palliative care and neurosurgery services can be added to those available to patients and their families at the HGT in the future.

## Conclusion

One of the critical factors for the long-term success of any collaborative transcultural program is to pursue healthy, long-term, and culturally sensitive relationships with government leaders and other stakeholders in the LMIC. Budgeting training and managerial skills are essential, as well as predictable and sustainable funding sources. Adherence to other Health Systems Strengthening principles enhances the likelihood of program success.

One of the short-term goals of all transcultural partnerships in pediatric oncology should be to improve clinical outcomes for children in LMICs. The long-term goal of all collaborative partnerships should be to transform an emerging pediatric oncology unit into an independent, high-quality, referral center that can initiate other local and regional collaborations.

There is a vast need for overcoming health disparities along the border region for both adults and children with various health conditions such as cardiac, infectious, metabolic, and surgical, among others; this represents a remarkable opportunity for improvement among clinicians, government officials, public health professionals, and other stakeholders on both sides of the border. The process that transcultural partnerships have developed to overcome health disparities is proven and could be applied to other disciplines of medicine.

Even within the HGT, where pediatric cancer care has already experienced dramatic improvements and where other physicians and departments have witnessed the success of this program, there are several opportunities to establish similar collaborations. Next steps could include engagement of key stakeholders on both sides of the border with a willingness to overcome any cultural and system barriers.

We invite pediatric cancer centers and academic institutions in LMIC and HIC to consider taking action regarding this ethical dilemma by initiating culturally sensitive, long-term collaborations with a sister organization in any discipline of medicine. We also encourage the enactment of policies that promote and facilitate such equitable, long-term transcultural partnerships.

## Author Contributions

PA, SF, RR, DB, RCR, and WR made substantial contributions to the conception, design, interpretation, and/or analysis of the project and data, the drafting and revising of the manuscript for important intellectual content, and the final approval of the published article.

## Conflict of Interest Statement

The authors declare that the research was conducted in the absence of any commercial or financial relationships that could be construed as a potential conflict of interest.

## References

[1] World Bank. World Development Indicators: Health Systems (2014). Available from: http://wdi.worldbank.org/table/2.15

[2] Howard SC Marinoni M Castillo L Bonilla M Tognoni G Luna-Fineman S Improving outcomes for children with cancer in low-income countries in Latin America: a report on the recent meetings of the Monza international school of pediatric hematology/oncology (MISPHO)-part I. Pediatr Blood Cancer (2007) 48(3):364–9. 10.1002/pbc.21003 16883601

[3] Howard SC Ribeiro RC Pui C Strategies to improve outcomes of children with cancer in low-income countries. Eur J Cancer (2005) 41(11):1584–7. 10.1016/j.ejca.2005.04.020 15979305

[4] Mostert S Sitaresmi MN Gundy CM Veerman AJ. Does aid reach the poor? Experiences of a childhood leukaemia outreach-programme. Eur J Cancer (2009) 45(3):414–9. 10.1016/j.ejca.2008.09.018 18977652

[5] Israels T Ribeiro RC Molyneux EM. Strategies to improve care for children with cancer in Sub-Saharan Africa. Eur J Cancer (2010) 46(11):1960–6. 10.1016/j.ejca.2010.03.027 20403685

[6] Delgado E Barfield RC Baker JN Hinds PS Yang J Nambayan A Availability of palliative care services for children with cancer in economically diverse regions of the world. Eur J Cancer (2010) 46(12):2260–6. 10.1016/j.ejca.2010.05.006 20541395 PMC2916078

[7] Kaatsch P Epidemiology of childhood cancer. Cancer Treat Rev (2010) 36(4):277–85. 10.1016/j.ctrv.2010.02.003 20231056

[8] Barr RD Klussmann FA Baez F Bonilla M Moreno B Navarrete M Asociación de hemato-oncología pediátrica de centro américa (AHOPCA): a model for sustainable development in pediatric oncology. Pediatr Blood Cancer (2014) 61(2):345–54. 10.1002/pbc.25143 24376230

[9] Farmer P Frenk J Knaul FM Shulman LN Alleyne G Armstrong L Expansion of cancer care and control in countries of low and middle income: a call to action. Lancet (2010) 376(9747):1186–93. 10.1016/S0140-6736(10)61152-X 20709386

[10] Masera G Baez F Biondi A Cavalli F Conter V Flores A North-South twinning in paediatric haemato-oncology: the La Mascota programme, Nicaragua. Lancet (1998) 352(9144):1923–6. 9863803 10.1016/s0140-6736(98)07077-9

[11] Rivera-Luna R Correa-González C Altamirano-Alvarez E Sánchez-Zubieta F Cárdenas-Cardós R Escamilla-Asian G Incidence of childhood cancer among Mexican children registered under a public medical insurance program. Int J Cancer (2013) 132(7):1646–50. 10.1002/ijc.27771 22886984

[12] Rivera-Luna R Shalkow-Klincovstein J Velasco-Hidalgo L Cárdenas-Cardós R Zapata-Tarrés M Olaya-Vargas A Descriptive epidemiology in Mexican children with cancer under an open national public health insurance program. BMC Cancer (2014) 14(1):790. 10.1186/1471-2407-14-790 25355045 PMC4228174

[13] Gupta S Rivera-Luna R Ribeiro RC Howard SC Pediatric oncology as the next global child health priority: the need for national childhood cancer strategies in low-and middle-income countries. PLoS Med (2014) 11(6):e1001656 10.1371/journal.pmed.1001656 24936984 PMC4061014

[14] St. Jude Children’s Research Hospital International Outreach Program. Guide to Establishing a Pediatric Oncology Twinning Program. Available from: https://www.stjude.org/SJFile/IOP_Twinning_Manual_082908.pdf

[15] Ribeiro RC Pui C Saving the children-improving childhood cancer treatment in developing countries. N Engl J Med (2005) 352(21):2158–60. 10.1056/NEJMp048313 15917380

[16] Pui C Ribeiro RC. International collaboration on childhood leukemia. Int J Hematol (2003) 78(5):383–9. 10.1007/BF02983810 14704030

[17] Veerman A Twinning: a rewarding scenario for development of oncology services in transitional countries. Pediatr Blood Cancer (2005) 45(2):103–6. 10.1002/pbc.20390 15920777

[18] Swanson RC Bongiovanni A Bradley E Murugan V Sundewall J Betigeri A Toward a consensus on guiding principles for health systems strengthening. PLoS Med (2010) 7(12):e1000385 10.1371/journal.pmed.1000385 21203584 PMC3006350

[19] Ries LG Smith MA Gurney JG Linet M Tamra T Young JL Cancer Incidence and Survival Among Children and Adolescents: United States SEER Program 1975–1995. Bethesda, MD: NIH Pub. No. 99-4649 (1999).

[20] Abdullaev FI Rivera-Luna R Roitenburd-Belacortu V Espinosa-Aguirre J. Pattern of childhood cancer mortality in Mexico. Arch Med Res (2000) 31(5):526–31. 10.1016/S0188-4409(00)00094-1 11179590

[21] Ribeiro RC Impact of the Mexican government’s system of social protection for health, or seguro popular, on pediatric oncology outcomes. Pediatr Blood Cancer (2013) 60(2):171–2. 10.1002/pbc.24355 23065960 PMC5114709

[22] Rodriguez-Galindo C Friedrich P Morrissey L Frazier L Global challenges in pediatric oncology. Curr Opin Pediatr (2013) 25(1):3–15. 10.1097/MOP.0b013e32835c1cbe 23295716

[23] Thompson JW Chesney RW Stocks RM Shmerling J Herron P. Pediatric hospitals’ and physician strategies for the 21st century. Clin Pediatr (1999) 38(5):259–63. 10.1177/000992289903800501 10349522

[24] Pérez-Cuevas R Doubova SV Zapata-Tarres M Flores-Hernández S Frazier L Rodríguez-Galindo C Scaling up cancer care for children without medical insurance in developing countries: the case of Mexico. Pediatr Blood Cancer (2013) 60(2):196–203. 10.1002/pbc.24265 22887842 PMC3561702

[25] Consejo Nacional de Evaluacion de la Politica de Desarollo Social. 2010 Poverty Levels for Each Municipality in Mexico (see: www.coneval.gob.mx) (2015). Available from: http://pressroom.ipc-undp.org/?p=8184

[26] Arenas F Salazar A Núñez A El aislamiento geográfico: ¿problema u oportunidad?: experiencias, interpretaciones políticas públicas. Santiago, Chile: Revista de Geografia Norte Grande (2011).

[27] Knight A Mexican national identity. In: Deans-Smith S Van Young E, editors. Mexican Soundings: In Honour of David A. Brading. Londres: Institute for the Study of the Americas (2007). p. 212–3.

[28] Williams EJ The resurgent north and contemporary mexican regionalism. Mex Stud (1990) 6(2):299–323. 10.2307/1051836

[29] Leavey PJ How many physicians do we need? A benchmark for pediatric hematology/oncology. Pediatr Blood Cancer (2013) 60(4):527–8. 10.1002/pbc.24396 23151998

[30] Goss PE Lee BL Badovinac-Crnjevic T Strasser-Weippl K Chavarri-Guerra Y St. Louis J Planning cancer control in Latin America and the Caribbean. Lancet Oncol (2013) 14(5):391–436. 10.1016/S1470-2045(13)70048-2 23628188

[31] Instituto Nacional de Estadística Geografía e Informática. Mexico. Available from: http://www.citypopulation.de/php/mexico-bajacalifornia.php

